# Genetic Evidence of Active Circulation and Evolution of Diverse Penguin Siadenoviruses in Antarctica Based on Partial DNA Sequences

**DOI:** 10.1155/tbed/5932514

**Published:** 2025-11-30

**Authors:** Sook-Young Lee, Sanghee Kim, Ji Hee Kim, Jong-U Kim, Jeong-Hoon Kim, Jihee Kim, Younggeun Oh, Jin-Won Song

**Affiliations:** ^1^Division of Life Sciences, Korea Polar Research Institute, Yeonsu-Gu, Incheon, Republic of Korea; ^2^Division of Life Sciences, Incheon National University, Yeonsu-Gu, Incheon, Republic of Korea; ^3^Department of Microbiology, College of Medicine, Korea University, Seongbuk-Gu, Seoul, Republic of Korea

## Abstract

Antarctica, one of the most isolated and extreme regions on Earth, hosts diverse bird species that share breeding and feeding habitats, facilitating interspecies transmission of pathogens. In this study, we investigated penguin siadenoviruses using cloacal swab samples collected from Antarctic penguins between 2017 and 2023 to explore their genetic diversity and evolutionary relationships. The complete hexon gene was obtained from Adélie penguins, while partial hexon and DNA polymerase sequences were detected in Adélie penguin (AP), chinstrap penguin (CP), and gentoo penguin (GP). Phylogenetic and molecular analysis identified multiple siadenoviruses classified into two distinct lineages, indicating ongoing viral evolution in this region. The hexon genes exhibited considerable genetic diversity caused by recombination and mutation, and predicted hypervariable regions (HVRs)—targets of neutralizing antibodies—showed significant structural differences among penguin siadenoviruses. These findings suggest that penguin siadenoviruses are not restricted to a single host species but may circulate among various penguin populations across the Antarctic region. This implies potential cross-infection between local and surrounding penguin populations. Although the predicted structural models showed limited accuracy due to the use of distant templates, the genetic and structural differences observed provide valuable insights into the adaptive evolution of these viruses. Our findings provide an important foundation for understanding viral transmission and evolution in Antarctic avifauna. Furthermore, findings from this study may guide early detection and risk assessment of emerging viral threats in Antarctica.

## 1. Introduction

Antarctica, the southernmost continent, is surrounded by the Antarctic Ocean and is the coldest, driest, and windiest continent [1]. Although the Antarctic region is geographically and climatically isolated, it is a habitat for various animals. Particularly, the Antarctic Peninsula and Ross Sea are representative habitats for many endemic animal species. The Antarctic Peninsula, located 1000 km (620 miles) from the southernmost tip of South America, is one of the most rapidly warming regions on Earth. Owing to its relatively mild climate and proximity to other continents, the Antarctic Peninsula experiences higher levels of biological connectivity and human activity than the rest of Antarctica. It serves as a critical habitat for a diverse range of avian and marine species, including penguins such as chinstrap penguins (CPs; *Pygoscelis antarcticus*), gentoo penguins (GPs; *P. papua*), and Adélie penguins (APs; *P. adeliae*) [2–4]. The Ross Sea region in southeastern Antarctica is an important ecological area. It is a habitat for endemic Antarctic animals and highly affected by climate and environmental changes. It is geographically located south of New Zealand and is relatively close to the southeastern coast of Australia, providing biological connectivity between Antarctica and Oceania. Additionally, it serves as the primary habitat and feeding area for numerous migratory animals, including seabirds and marine mammals, some of which migrate seasonally across temperate regions in the subantarctic region and southern hemisphere [5]. Notably, large breeding colonies of AP exist in the Ross Sea region [6]. Antarctic animals have ecological features, including the global migration by some birds and habitat sharing involving close contact, which could serve as potential pathways for the exchange of pathogens, including viruses, between the Antarctic region and subantarctic ecosystems. Furthermore, both regions function as key gateways for the potential emergence of pathogens through bird migration and increasing anthropogenic activities such as research operations and tourism [7–9]. Antarctica has long been considered an infectious virus-free region owing to its geographical and climatic isolation. However, the emergence and spread of infectious viruses in Antarctica has increased recently [9]. For example, various subtypes of avian paramyxoviruses have been isolated from penguins in the Antarctic Peninsula, and several subtypes of low-pathogenic avian influenza virus (LPAIV) have been detected in Antarctic penguins [10–15]. Notably, various subtypes of LPAIVs have also been found repeatedly within Antarctic penguin populations since they were first detected in 2013 and have been found to circulate in Antarctica, forming a genetically distinct single cluster [13]. Additionally, penguin adenoviruses have been detected in penguin populations since the first report in 2008 up to 2013 [16, 17]; recent findings have indicated that they have continued to circulate up to 2023 [18]. Several factors play a role in the emergence and transmission of viruses. For instance, Antarctic animals can serve as efficient carriers of viruses, and close interactions among them can contribute to the spread of the viruses [7, 8]. Therefore, continuous monitoring is required to understand the transmission and circulation of viruses in the Antarctic ecosystem, enabling early detection of adverse impacts and potential outbreaks.

Adenoviruses (family: Adenoviridae) are linear, double-stranded DNA viruses with 26–45 kbp genome size and icosahedral capsid [19]. Adenoviruses contain three major capsid proteins: the penton base, hexon, and fiber [20–22]. The penton base contributes to several steps in the early stages of infection, including cell surface binding and receptor-mediated endocytosis. The fiber consists of a rod and knob attached to the penton base, which contributes considerably to the binding ability and infectivity of the virus [21]. The hexon is a major component of the capsid. It forms trimers and capsomers and plays a central role in inducing humoral, cellular, and innate immune responses, as well as determining the serotype [23]. Adenoviral hexon is composed of three regions from the base to the top. Notably, the top of the molecule contains loop domains (DE1, FG1, and FG2) that form the outer surface of the viral capsid. This region contains hypervariable regions (HVRs), which are crucial for determining the serotype and are involved in interactions with the host immune system. These HVRs are surface-exposed loops of hexon proteins that vary greatly among different adenovirus serotypes. This variability makes the HVRs key targets for neutralizing antibodies and influences the ability of viruses to infect cells and evade immune responses [23–25]. Adenoviral DNA polymerase, a major functional protein, is responsible for replicating the adenoviral genome. It is part of a multiprotein complex that binds to the origin of replication [26]. Adenoviruses infect a diverse array of hosts, ranging from fish to humans. The Adenoviridae family comprises six genera classified according to their hosts: *Mastadenovirus*, *Aviadenovirus*, *Barthadenovirus*, *Siadenovirus*, *Ichtadenovirus*, and *Testadenovirus* [19, 27]. Mastadenoviruses have been identified in mammals, including humans, and are known to cause respiratory infections, conjunctivitis, and gastrointestinal infections. Barthadenoviruses have been found in reptiles, birds, and ruminants and can cause egg drop syndrome in chickens and severe disease in some species. Ichtadenoviruses and testadenoviruses have only been found in fish and tortoises, with few known disease cases. Aviadenoviruses, found in birds, are considered an important pathogen in the poultry industry because they cause a variety of diseases. Siadenoviruses are mainly found in birds and amphibians and are known to cause respiratory diseases and systemic infections in birds [28]. Infection with turkey adenovirus-3 (TAdV-3), a siadenovirus, causes severe symptoms, including depression, splenomegaly, hemorrhagic diarrhea, and immunosuppression, and death in turkeys [29–31]. In 2013, a novel species of *Siadenovirus*, which was genetically similar to TAdV-3, was discovered in CPs and GPs. These novel penguin siadenoviruses were found to possess a unique genetic structure characterized by the deletion of the *sialidase* gene [17]. Building upon the discovery of these novel penguin siadenovirus, our current study reports the discovery of additional siadenoviruses in Antarctic penguins and provides a detailed analysis of their genetic characteristics as part of the ongoing viral disease monitoring.

## 2. Materials and Methods

### 2.1. Samples

**Figure 1:**
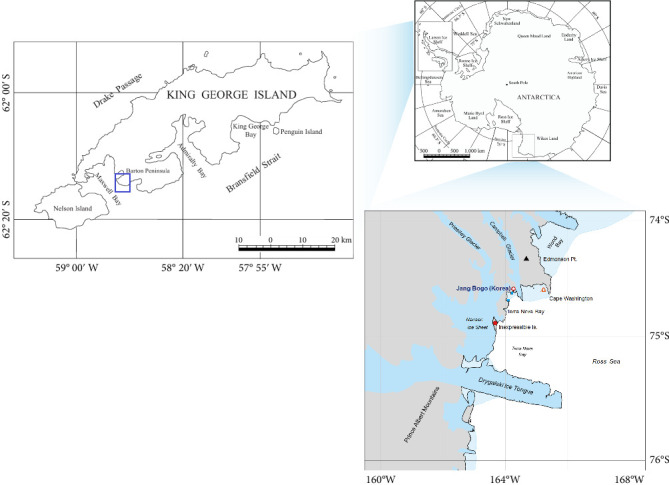
Collection sites of cloacal swab samples of penguins. The left side was Barton Peninsula, King George Island in the Antarctic Peninsula, and the lower side was Inexpressible Island, Terra Nova Bay in South Antarctica. The Barton Peninsula, where samples of chinstrap penguins and gentoo penguins were collected, and Inexpressible Island, where samples of Adelie penguins were collected, are marked with blue squares.

Cloacal swab samples of AP were collected at Inexpressible Island (ASPA No. 178), Terra Nova Bay, in the Ross Sea, in December 2023. Additionally, cloacal swab samples of GP and CP were collected at Narıbski Point (ASPA No. 171), King George Island, West Antarctica, from 2017 to 2023 (Figure 1). All samples were collected in transport medium and stored at −20°C until use for adenovirus identification. This study also involved the analysis of the hexon and DNA polymerase sequences of penguin siadenoviruses (CP10-4, CP10-6, GP10-6, and GP10-7), which were found in samples collected in 2010 but were not reported previously (Supporting Information 1: Table S1). All animal experiments were conducted in accordance with institutional guidelines and were approved by the Animal Ethics Committee of the Korea Polar Research Institute (Approval number KACC2202-005 and KACC2301-024).

### 2.2. Polymerase Chain Reaction (PCR) and DNA Sequencing

Total DNA was extracted from 200 µL of cloacal swab samples using the AllPrep PowerViral DNA/RNA kit (QIAGEN, Germany), according to the manufacturer's instructions. For adenovirus screening, positions 2196–2704 nt of the hexon gene and positions 1165–1664 nt of the DNA polymerase (*pol*) gene were targeted, while the complete hexon gene sequence was obtained for sequence analysis [17]. PCRs were performed under the following conditions: 98°C for 2 min, followed by 40 cycles consisting of denaturation at 98°C for 30 s, annealing at 45°C/50°C for 30 s (hexon/pol), and extension at 72°C for 1 min and at 72°C for 5 min. These were performed in a Mastercycler (Eppendorf, Germany). The amplified products were purified using a PCR Purification Kit (QIAGEN, USA) and sequenced using BigDye 3.1 terminator cycle sequencing reagents on an ABI 3730 Automated DNA Sequencer (Applied Biosystems, USA).

### 2.3. Phylogenetic Analysis

Phylogenetic analysis was performed based on the partial hexon and DNA polymerase genes. Reference sequences for sequence comparison were retrieved from GenBank (Supporting Information 1: Table S1). Multiple sequence alignments (MSAs) of the adenoviral sequences were generated using the ClustalW method in BioEdit version 7.0.9.0. The topologies of maximum likelihood trees were generated with bootstrap analysis of 1000 iterations using MEGA10.1.7 software [32].

### 2.4. Protein Structure Modeling and Comparative Analysis

Since the hexon protein structure of penguin siadenovirus and related siadenoviruses has not yet been revealed, the hexon proteins of penguin siadenoviruses were simulated using AlphaFold2 (ColabFold) and homology modeling on a SWISS-MODEL online server based on the complete hexon amino acid sequences [33–35]. The template for modeling the hexon protein structure was searched on the server and selected by comparing it with the results of the amino acid sequence homology from MMseqs2 and HHblits, respectively. In SWISS-MODEL, the selection criteria for the template were determined based on the quality of the resulting models, as estimated by the global model quality estimate (GMQE), quaternary structure quality estimate (QSQE), and sequence identity. The models generated by AlphaFold2 were evaluated and selected based on predicted local distance difference test (pLDDT) scores, MSA coverage, and predicted aligned error (PAE), while those generated by SWISS-MODEL were selected according to the qualitative model energy analysis (QMEAN) scores, which indicated the model accuracy. All structures were visualized using the PyMOL3.1 program [36].

The protein structures were compared using pairwise structure alignment on the RCSB webserver [37], and the simulated hexon protein structures of penguin siadenoviruses were used for this analysis. Structural differences were expressed as root mean square deviation (RMSD) and template modeling (TM) scores, both of which are metrics used to assess similarity between two protein structures. While a lower RMSD value indicates a better structural match between the two proteins, a TM score closer to 1 indicates a better structural match [38].

### 2.5. Genetic Recombination

Recombination in the penguin adenoviral hexon genes was evaluated using RDP, GENECONV, Bootscan, MaxChi, Chimaera, Siscan, and three sequencing methods implemented in RDP5.67 with the following general recombination detection options: sequences set to linear, Bonferroni correction, and the highest acceptable *p*-value set to 0.05. This was visualized using a distance plot [39].

## 3. Results

### 3.1. Adenovirus Detection

Nine adenoviral sequences (five siadenoviruses) were obtained from the cloacal swab samples of the five Antarctic penguins (one GP, two CPs, and two APs). Adenoviral partial hexon genes were detected in GP17-6, CP23-5, and CP23-6, whereas partial DNA polymerase genes were detected in GP17-6, CP23-5, CP23-6, and AP23-9. The complete adenoviral hexon sequences were obtained from AP23-8 and AP23-9. However, no DNA polymerase gene sequences were detected in strain AP23-8 (Supporting Information 1: Table S1).

### 3.2. Phylogenetic Analysis of the Hexon Gene

**Figure 2:**
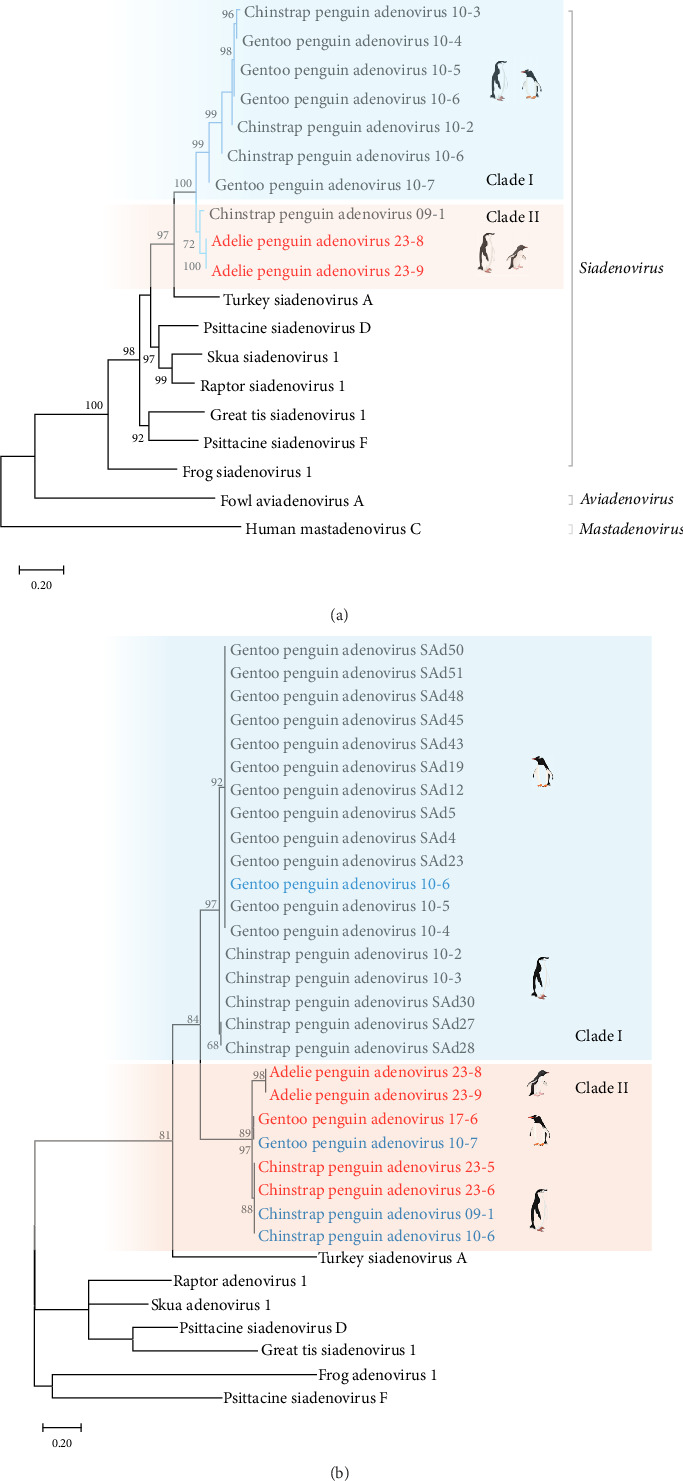
Phylogenetic trees of Antarctic penguin siadenoviruses. Phylogenetic trees of complete hexon genes (A) and partial hexon genes (amino acid position 716–825, 109 aa) (B) of penguin adenoviruses and other siadenoviruses. Penguin siadenoviruses discovered in this study and unpublished penguin siadenoviruses are indicated in bold red and blue, respectively.

The phylogenetic tree of the complete adenoviral hexon sequences detected in Antarctic penguins showed that these viruses were divided into two independent clades (Figure 2A). The genetic distances between all penguin siadenoviruses were 0%–17.1%. These penguin siadenoviruses showed the closest similarity to TAdV, a different species of the *Siadenovirus* genus, with distances of 21%–23.5%. Notably, the hexon genes of AP siadenovirus (APAdV23-8 and APAdV23-9) discovered on Inexpressible Island in 2023 showed a distinct clade from previously reported penguin adenoviral hexon genes with high bootstrap values and exhibited genetic differences of 7.5%–17.1%. The hexon genes of APAdV23-8 and APAdV23-9 were most similar to those of CPAdV09-1, which was found in the Fields Peninsula in 2009. Although GPAdV10-7 was clustered in clade Ⅰ (89.3%–90.9% similarity with others), it showed higher similarity to penguin siadenoviruses in clade Ⅱ (91.3%–92.5%), except for CPAdV10-6 within clade Ⅰ (94.6%). Phylogenetic analysis based on the partial hexon sequences of penguin siadenoviruses, including the FG2 region, revealed two clades (Figure 2B).

In addition to the previously published penguin siadenoviruses (GPAdV10-7 and CPAdV09-1), newly identified viruses, such as GPAdV17-6, CPAdV23-5, CPAdV23-6, APAdV23-8, and APAdV23-9, were classified into clade Ⅱ, showing genetic distances of 27.4%–32% from those in clade Ⅰ. Within clade II, the sequence distances were 0%–8.7%, and APAdV23-8 and APAdV23-9 showed differences of 7.6%–8.7% in the same clade. In the phylogenetic tree of the partial hexon gene, clades I and II showed host species-associated clustering within each clade.

### 3.3. Phylogenetic Analysis of the DNA Polymerase Gene

**Figure 3:**
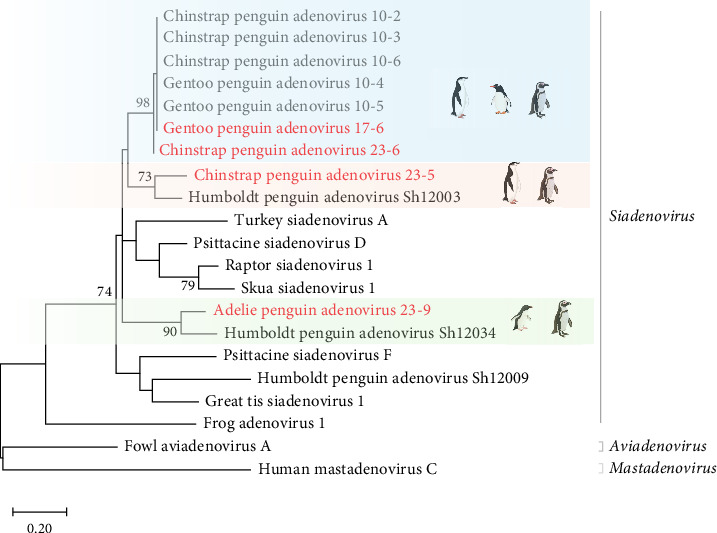
Phylogenetic tree of partial DNA polymerase gene of Antarctic penguin siadenoviruses. Penguin siadenoviruses discovered in this study are indicated in bold red. The sequences used in the analysis correspond to amino acid positions 593–682 (89 aa).

Phylogenetic analysis of the partial DNA polymerase showed that GPAdV17-6 and CPAdV23-6 clustered with the DNA polymerase sequences of previously reported penguin siadenoviruses with high bootstrap values. The sequence identity of this cluster was 99.9%–100%. Contrastingly, the partial DNA polymerase sequences of CPAdV23-5 showed the highest similarity of 83.1% to Humboldt penguin siadenovirus sequences discovered in Peru (Sh12003, ATY47788) and 69.7%–77.5% genetic similarity to previous Antarctic penguin siadenovirus sequences. Additionally, the partial DNA polymerase sequence from APAdV23-9 found on Inexpressible Island had the highest similarity (82%) with Humboldt penguin siadenovirus sequences (Sh12034, ATY47789) and had 74.2% similarity with the cluster of previously reported Antarctic penguin siadenoviruses (Figure 3).

### 3.4. 3D Modeling and Comparison of Hexon Proteins of Penguin Adenovirus

Adenoviral hexon proteins of Antarctic penguins were modeled using the complete hexon amino acid sequences of three genetically distinct viruses: CPAdV10-2, CPAdV10-6, and APAdV23-9. The hexon protein predicted via AlphaFold2 selected the most reliable model (rank1), and only protein regions with pLDDT scores greater than 50 were used for analysis (Supporting Information 1: Table S2). On the other hand, in protein prediction by SWISS-MODEL, based on GMQE, QSQE, and sequence identity, the hexon proteins of Lizard adenovirus 2 (6qi5) and Fowl adenovirus C (8roq) with the highest scores were selected as templates for modeling the hexon protein structures of the penguin siadenovirus. As a result of modeling based on the two templates, three hexon proteins, CPAdV10-2, CPAdV10-6, and APAdV23-9, based on Fowl adenovirus C (8roq) were finally generated with higher QMEAN scores (Supporting Information 2: Figure S1; Supporting Information 1: Table S3).

**Figure 4:**
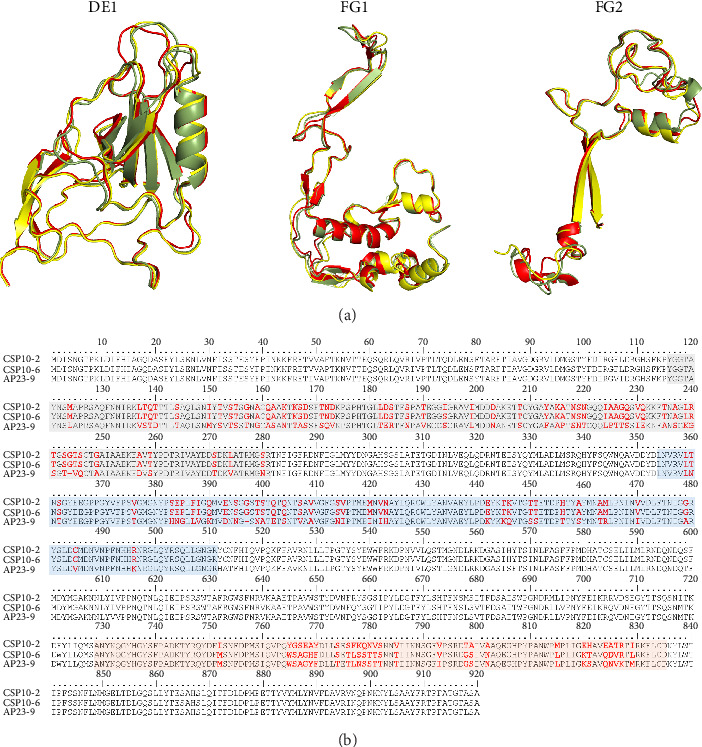
Comparison of hypervariable regions (DE1, FG1, and FG2) of penguin siadenovirus hexon proteins. (A) Comparison of expected DE1, FG1, and FG2 regions of CPAdV10-2 (green), CPAdV10-6 (yellow), and APAdV23-9 (red) hexon proteins predicted by AlphaFold2. (B) The amino acid alignment of three hexon genes. The gray, blue, and orange squares in the amino acid alignments represent the putative DE1, FG1, and FG2 regions, respectively. The bold red amino acids indicate differences between the three hexon proteins.

To compare protein structures, the hexon proteins of the three penguin siadenoviruses generated by AlphaFold2 were aligned. As shown in Figure 4A, structural differences were observed in the DE1, FG1, and FG2 regions. The DE1, FG1, and FG2 regions of the three adenoviral hexons showed significant differences in amino acid sequences (Figure 4B). Comparison of the amino acid sequences of these regions revealed that the DE1 region of APAdV23-9 showed 33.9% and 36.5% differences to those of CPAdV10-6 and CPSAdV10-2, respectively. Additionally, the FG1/2 regions of APAdV23-9 differed from those of the two penguin siadenoviral hexons by 22.3%, 9.3%, 22.3%, and 26.2% (Table 1). Comparison of the RMSD and TM score between each region of the three proteins revealed that APAdV23-9 showed an RMSD of 0.91–1.95 and a TM score of 0.8–0.97 with the other penguin siadenoviruses (Supporting Information 1: Table S4).

### 3.5. Intergenic Recombination Between Antarctic Penguin Adenoviruses

**Figure 5:**
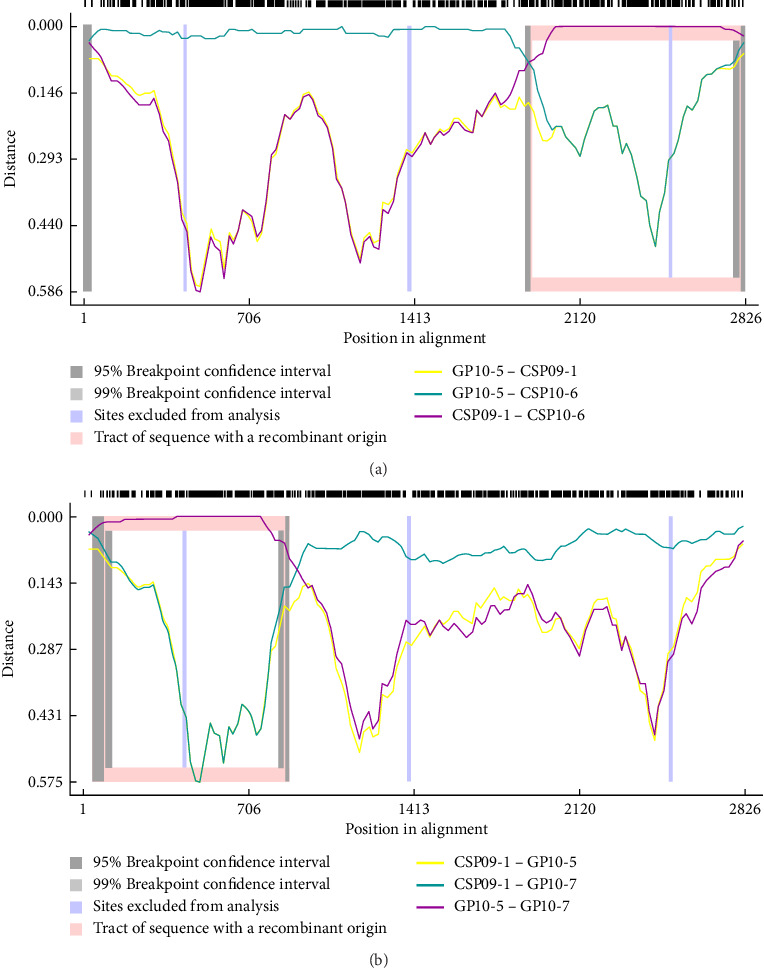
Genetic recombination between the Antarctic penguin siadenoviruses. (A) Recombination event of CPAdV10-6. (B) Recombination event of GPAdV10-7.

Based on the results of phylogenetic analyses of the complete hexon and partial hexon sequences, recombination events between penguin siadenoviruses were evaluated. Two recombination events were confirmed in the hexon genes CPAdV10-6 (*p*= 1.78e-83–1.20e-30) and GPAdV10-7 (*p*=2.19e-68–2.44e-29). The hexon gene of CPAdV10-6 was predicted to have undergone recombination with CPAdV09-1 (clade Ⅱ) at a site located downstream of approximately amino acid position 1872. The hexon gene of GPAdV10-7 was predicted to have recombined with GPAdV10-5 (clade Ⅰ) at a site upstream of approximately amino acid position 866. However, the major parents of GPAdV10-7 and CPAdV10-6 hexons were CPAdV09-1 (clade II) and GPAdV10-5 (clade I), respectively (Figure 5).

## 4. Discussion

Studies on Antarctic viruses are limited because of the geographical isolation of Antarctica and the limited awareness of its viral ecology. However, due to interest in zoonoses following outbreaks of novel infectious viruses and concerns about climate change, research on infectious viruses in Antarctic animals is gaining momentum. Following the discovery of a novel species of penguin siadenovirus in Antarctica in 2013, adenoviruses have been periodically monitored using cloacal swab samples collected from penguins in Western and Eastern Antarctica between 2017 and 2023. In this study, we detected adenoviral hexon and DNA polymerase genes in five Antarctic penguins: one GP (GP17-6), two CPs (CP23-5 and 23-6), and two APs (AP23-8 and 23-9). These viral sequences were genetically more similar to previously reported Antarctic penguin siadenoviruses (CPAdV10-2, 3, GPAdV10-4, and 10-5) than to other siadenoviruses. Analysis of the complete and partial hexon genes showed that, although monophyletic, the hexon genes of penguin siadenoviruses were genetically divided into two clades. It appears that penguin siadenoviruses share a common ancestor and that they have evolved into two indigenous lineages in Antarctica. Particularly, the complete hexon genes of APAdV23-8 and APAdV23-9 were more closely related to those of CPAdV09-1 than to those of any other known penguin siadenovirus. Despite spatiotemporal differences in the sampling of APAdV23-8, APAdV23-9, and CPAdV09-1, viruses belonging to the independently evolved clade II may have undergone interspecies transmission. The Barton Peninsula and Inexpressible Island are ~4500 km apart, but APs are distributed throughout Antarctica and migrate up to 17,600 km [40, 41]. This suggests that penguin siadenoviruses might have been spread through contact with other penguin species during APs' migration. The Antarctic penguin siadenoviruses discovered so far were not genetically diverse because all partial hexon sequences were similar. However, the present study confirmed the presence of genetically diverse siadenoviruses in Antarctic penguins. Moreover, the different phylogenetic locations of CPAdV10-6 and GPAdV10-7 in the phylogeny of complete and partial hexons implied that the specific hexon regions of these viruses originated from other viruses. Recombination analysis revealed that GPAdV10-7 originated from CPAdV09-1 of clade II as the major parent but underwent genetic recombination with GPAdV10-5 of clade I in specific regions (*p*=2.19e-68–2.44e-29). Similarly, the hexon gene of CPAdV10-6 appears to have originated from GPAdV10-5 of clade I but shows evidence of recombination with CPAdV09-1 of clade II (*p*=1.78e-83–1.20e-30). This supports the hypothesis that a genetic exchange occurred between the siadenoviruses of clades I and II. The ecological environment of Antarctica features densely populated penguin colonies, which promotes frequent physical contact between penguins and allows viruses to spread easily. Moreover, the low-temperature Antarctic environment provides favorable conditions for prolonged viral survival of the virus even in habitats contaminated with penguin excreta, thereby facilitating its transmission and persistence [42–44]. Visits from migratory birds from different continents and their interactions with other animals can diversify the routes of viral emergence. These factors facilitate viral transmission between different animal species in Antarctica and enable genetic recombination between different viruses.

Recombination and mutation in adenoviruses can lead to changes in viral characteristics such as replication, preferred host, and antigenicity. Particularly, hexon, a major component of capsids, is known to play an important role in inducing humoral, cellular, and innate immune responses and determining serotypes [21–23, 45]. The top of the monomeric hexon molecule forms the outer surface of the viral capsid upon exposure to loop domains DE1, FG1, and FG2. Neutralizing epitopes of adenoviruses are located in these domains [24, 25]. Penguin siadenoviruses found in Antarctica are predicted to exhibit structurally distinct differences in these domains. Notably, APAdV23-9 showed significant differences in the amino acid sequences and protein structures of each domain from CPAdV10-2 and CPAdV10-6. In previous reports, the sequence differences between DE1, FG1, and FG2 within the family Adenoviridae were 54%, 29%, and 28%, respectively. Furthermore, the differences in the complete hexon amino acid sequences among the 51 serotypes of human adenoviruses were 0.7%–25.7% [25, 46]. However, there were differences of 13%–16.3% between the complete hexon amino acids of APAdV23-9, CPAdV10-2, and CPAdV10-6; the differences in amino acid sequences and the RMSD values of predicted protein structures in the three domains were 9.3%–36.5% and 0.91–1.95, respectively. Despite the limited accuracy of the predicted structures and the instability of loop predictions in the protein structure, these genetic differences in the epitope regions may imply structural differences between APAdV23-9 and other penguin siadenoviruses belonging to clade I [47, 48]. Genetic differences in epitope regions can manifest as serological differences due to structural changes in proteins. Moreover, serological differences can lead to the appearance of new pathotypes through evasion of the host immune response. However, since viruses with different genotypes within the serotype can still share antigenic properties and the immune status of hosts is different, the genotype, serotype, and pathotype of the viruses are not always perfectly correlated [49, 50]. Therefore, the serological interactions and pathogenicity depending on the genotype of Antarctic penguin siadenoviruses require further studies.

Partial DNA polymerase genes show unique genetic differences. The partial DNA polymerase genes of GPAdV17-6 and CPAdV23-6 were similar to those of previously reported penguin siadenoviruses, whereas APAdV23-9 and CPAdV23-5 showed higher genetic similarity with Humboldt penguin siadenoviruses reported in Peru [44]. However, the amino acids of the partial DNA polymerase genes of APAdV23-9 and CPAdV23-5 differed by 18% and 16.9%, respectively, from those of the Humboldt penguin. Since this amino acid difference is based on a partial DNA polymerase sequence, further analyses—such as obtaining the complete DNA polymerase sequence, examining nucleotide composition, and investigating genome organization—will be necessary to confirm whether APAdV23-9 and CSPAdV23-5 are novel siadenoviruses [19, 28]. The Humboldt penguin siadenovirus has been reported only from a partial DNA polymerase sequence. Given the limited number of viral sequences and the reliance on partial sequences, inferring a potential transmission relationship between Antarctic and Humboldt penguin siadenoviruses is challenging [16, 18, 44]. Furthermore, considering the distinct and non-overlapping geographic distributions of Antarctic and Humboldt penguins, direct transmission between these species appears unlikely [51]. Instead, other penguin species, such as Magellanic and King penguins, which inhabit regions between Antarctica and the range of Humboldt penguins, may act as ecological intermediaries bridging this geographic gap [52, 53]. Most of the available data on siadenoviruses originating from Antarctica are partial sequences; therefore, it is very difficult to obtain a lot of information. Additional information, particularly adenovirus sequences from other penguin species, is needed to fill the knowledge gaps on the genetic differences between penguin siadenoviruses and Humboldt penguin siadenoviruses. Whole-genome data and long-term monitoring of viruses can provide better insights into evolutionary variations and transmission of the viruses.

## 5. Conclusions

Currently, Antarctic penguin siadenoviruses have only been reported in CPs and GPs. However, this study also identified siadenoviruses in APs. The newly identified viruses showed genetic differences in the complete hexon and partial DNA polymerase gene compared with previously known siadenoviruses. Despite limitations in structural modeling, these genetic and structural differences indicate active circulation and ongoing evolution of diverse penguin siadenoviruses. Our phylogenetic and molecular analyses provide insights into viral adaptation and potential host range expansion. These findings underscore the importance of monitoring penguin siadenoviruses to better understand viral evolution and interspecies transmission in Antarctic bird populations.

## Figures and Tables

**Table 1:** Comparison of amino acid sequences of the hexon loop regions (DE1, FG1, and FG2) of three penguin adenoviruses.

Region	Sequence similarity (distance %)		
DE1	CSP10-2^a^	CSP10-6	AP23-9
CSP10-2^a^	—	99.4% (0.6%)	63.5% (36.5%)
CSP10-6	99.4% (0.6%)	—	66.1 (33.9%)
FG1	CSP10-2^a^	CSP10-6	AP23-9
CSP10-2^a^	—	100% (-)	77.7% (22.3%)
CSP10-6	100% (-)	—	77.7% (22.3%)
FG2	CSP10-2^a^	CSP10-6	AP23-9
CSP10-2^a^	—	77.6% (22.4%)	73.8% (26.2%)
CSP10-6	77.6% (22.4%)	—	90.7% (9.3%)

^a^Reference adenovirus strain (ABL78138).

## Data Availability

The sequences of hexon and DNA polymerase genes have been deposited in the GenBank under accession number PX453681–PX453689.

## Supporting Information

Supporting Information 1 Table S1: Adenoviruses detected inn Antarctic birds and reference adenovirus used for analysis in this study. Table S2: Average pLDDT scores for each hypervariable region of the hexon protein predicted by AlphaFold2. Table S3: Values calculated by SWISS-MODEL for simulation of the hexon protein structures. Table S4: Measurement of protein structure similarity in DE1, FG1, and FG2 regions (RMSD/TM-score)

Supporting Information 2 Figure S1 Comparison of complete hexon proteins of penguin siadenoviruses generated by SWISS-MODEL. (A) 3D modeling of complete hexon proteins of CPAdV10-2, CPAdV10-6, and APAdV23-9 based on the hexon protein of fowl adenovirus C4 (8roq). The 3D models using three adenoviral hexons were aligned, and expected DE1, FG1, and FG2 regions of hexon proteins are marked in red, yellow, and orange, respectively. (B) Comparison of the expected DE1, FG1, and FG2 regions of hexon proteins of CPAdV10-2 (green), CPAdV10-6 (yellow), and APAdV23-9 (red).
