# Supplementary figures and images for: Genetic Evidence of Active Circulation and Evolution of Diverse Penguin Siadenoviruses in Antarctica Based on Partial DNA Sequences
Source: Transbound Emerg Dis. 2025 Nov 30;2025:5932514. doi: 10.1155/tbed/5932514 (PMC12682450; doi:10.1155/tbed/5932514)

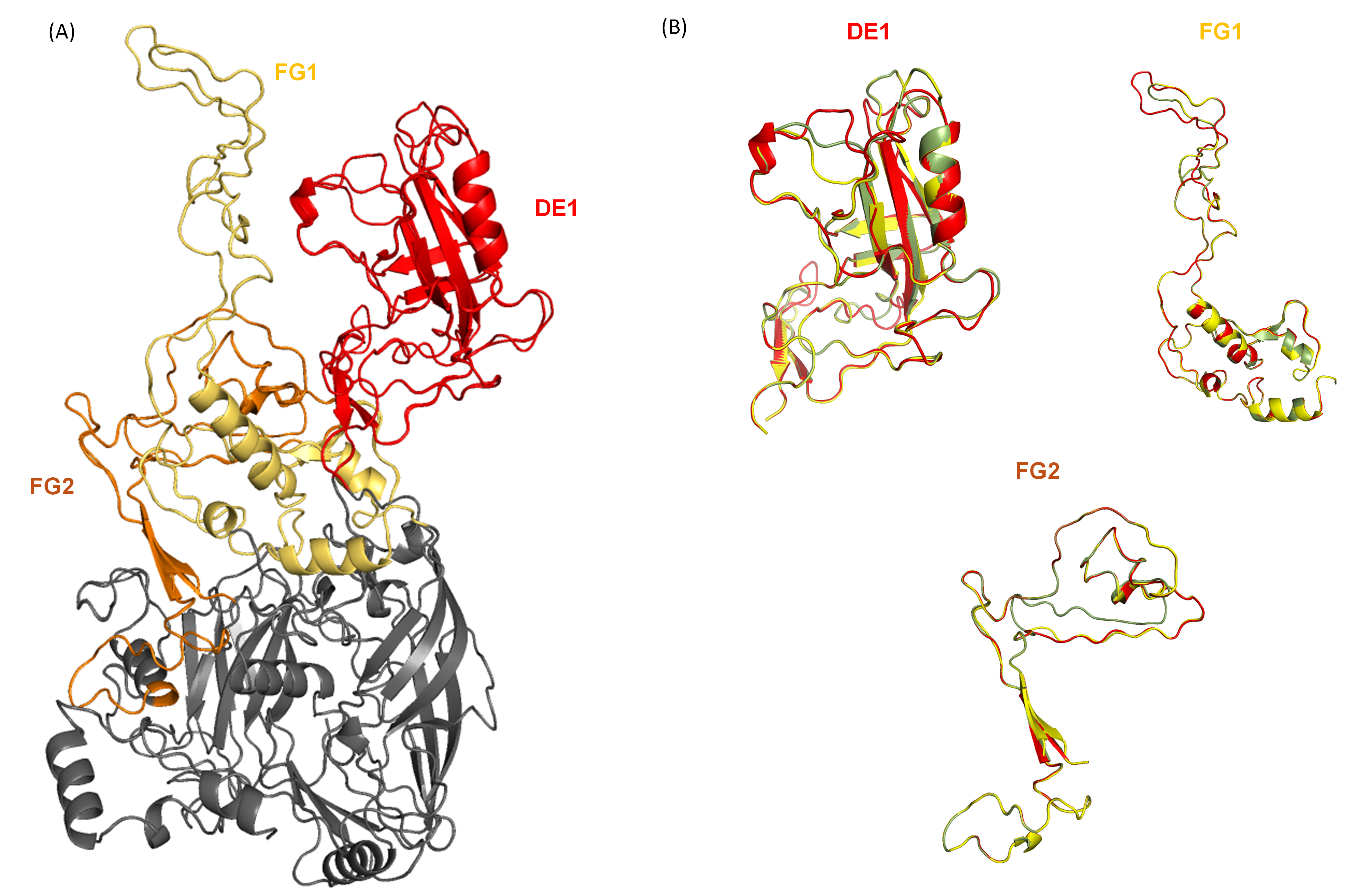

Supplement: Supporting Information 1 — Table S1: Adenoviruses detected inn Antarctic birds and reference adenovirus used for analysis in this study. Table S2: Average pLDDT scores for each hypervariable region of the hexon protein predicted by AlphaFold2. Table S3: Values calculated by SWISS-MODEL for simulation of the hexon protein structures. Table S4: Measurement of protein structure similarity in DE1, FG1, and FG2 regions (RMSD/TM-score) [file 5932514.f1.png]
